# Unrepaired decompressive craniectomy worsens motor performance in a rat traumatic brain injury model

**DOI:** 10.1038/s41598-020-79155-8

**Published:** 2020-12-17

**Authors:** Brian T. Andrews, Scott Barbay, Jakob Townsend, Michael Detamore, Janna Harris, Chad Tuchek, Randolph J. Nudo

**Affiliations:** 1 Department of Plastic and Reconstructive Surgery, University of Kansas Medical Center, Sutherland Institute, MS 3015, 3901 Rainbow Blvd, Kansas City, KS 66160 USA; 2 Department of Rehabilitation Medicine, University of Kansas Medical Center, Kansas City, KS USA; 3 School of Biomedical Engineering, University of Oklahoma, Norman, OK USA; 4 Department of Anatomy and Cell Biology, University of Kansas, Kansas City, KS USA; 5 Department of Neurosurgery, University of Kansas Medical Center, Kansas City, KS USA

**Keywords:** Neuroscience, Medical research

## Abstract

Decompressive craniectomy (DC) is often required to manage rising intracranial pressure after traumatic brain injury (TBI). Syndrome of the trephine (SoT) is a reversible neurologic condition that often occurs following DC as a result of the unrepaired skull. The purpose of the present study is to characterize neurological impairment following TBI in rats with an unrepaired craniectomy versus rats with a closed cranium. Long Evans male rats received a controlled cortical impact (CCI) over the caudal forelimb area (CFA) of the motor cortex. Immediately after CCI, rats received either a hemi-craniectomy (TBI Open Skull Group) or an immediate acrylic cranioplasty restoring cranial anatomy (TBI Closed Skull Group). Motor performance was assessed on a skilled reaching task on post-CCI weeks 1—4, 8, 12, and 16. Three weeks after the CCI injury, the TBI Closed Skull Group demonstrated improved motor performance compared to TBI Open Skull Group. The TBI Closed Skull Group continued to perform better than the TBI Open Skull Group throughout weeks 4, 8, 12 and 16. The protracted recovery of CFA motor performance demonstrated in rats with unrepaired skulls following TBI suggests this model may be beneficial for testing new therapeutic approaches to prevent SoT.

## Introduction

Decompressive craniectomy (DC) is a neurosurgical procedure utilized to treat severe traumatic brain injury (TBI). This procedure, popularized by Cushing in 1908 to treat brain injuries associated with depressed skull fractures, involves the removal of a large segment of cranial bone at the site of brain injury^1,2^. DC mitigates rising intracranial pressure, thus preventing cortical brain injury by allowing the brain to swell and herniate beyond the confines of the rigid cranial vault. Eventually, the cranial bone is restored/replaced in a second operation, termed cranioplasty^3^.

Ideally, cranioplasty is performed two to four weeks following DC to avoid a “sinking skin flap syndrome” appearance as the unsupported scalp skin begins to compress the underlying brain. However, cranioplasty commonly does not occur for weeks to months following DC^4,5^. As a result, neurorehabilitation is often delayed by “syndrome of the trephined” (SoT)^6^. SoT is a poorly understood neurologic condition that occurs in patients with unrepaired cranial bone defects in conjunction with sinking skin flap syndrome. SoT is manifested by a variety of symptoms that include: fine motor dexterity concerns, headaches, feelings of anxiety, apprehension, depression, and difficulties concentrating^7,8^. SoT symptoms are generally independent of TBI location and mechanism. Size of the cranial defect (> 50 cm^2^) is most predictive of SoT symptom manifestation^9^. Interestingly, the symptoms of SoT are reversible once the cranial vault anatomy is restored either by replacing the cranial bone or using an implant to reconstruct the cranial defect weeks to months after the initial TBI^3^.

Although clinical experience documents SoT well, large series publications describing this syndrome are limited, and few studies have investigated it in laboratory animals. Cranioplasty immediately following DC has been demonstrated to reverse motor deficits in a mouse model using a beam walk assessment^10^. In addition, increased apprehension and anxiety are significantly demonstrated in rats that undergo hemi-craniectomy independent of TBI. The aim of the present study is to compare neurobehavioral motor deficits between DC and immediate cranioplasty following open TBI. Our hypothesis is that maintaining “normal” cranial vault anatomy is an important independent factor in determining TBI outcomes.

## Methods

### Animal subjects

Study approval was granted by the Institutional Animal Care and Use Committee at the University of Kansas Medical Center (#2018-2440). Ten-week-old male Long Evans rats (370 ± 18.8 g at the time of the surgery) were obtained from Envigo (Summerset, NJ). Rats were housed in pairs upon arrival for 1–2 weeks and allowed to acclimate at 22° C with a 12 h/12 h light–dark cycle prior to the study initiation. Rats were handled daily during the acclimation period and food and water were provided ad libitum. Rat were then individually housed at the start of behavioral training through the duration of the study. All animal utilization was done in accordance with the National Institutes of Health regulations.

### Controlled cortical impact and craniectomy/repair

**Figure 1:**
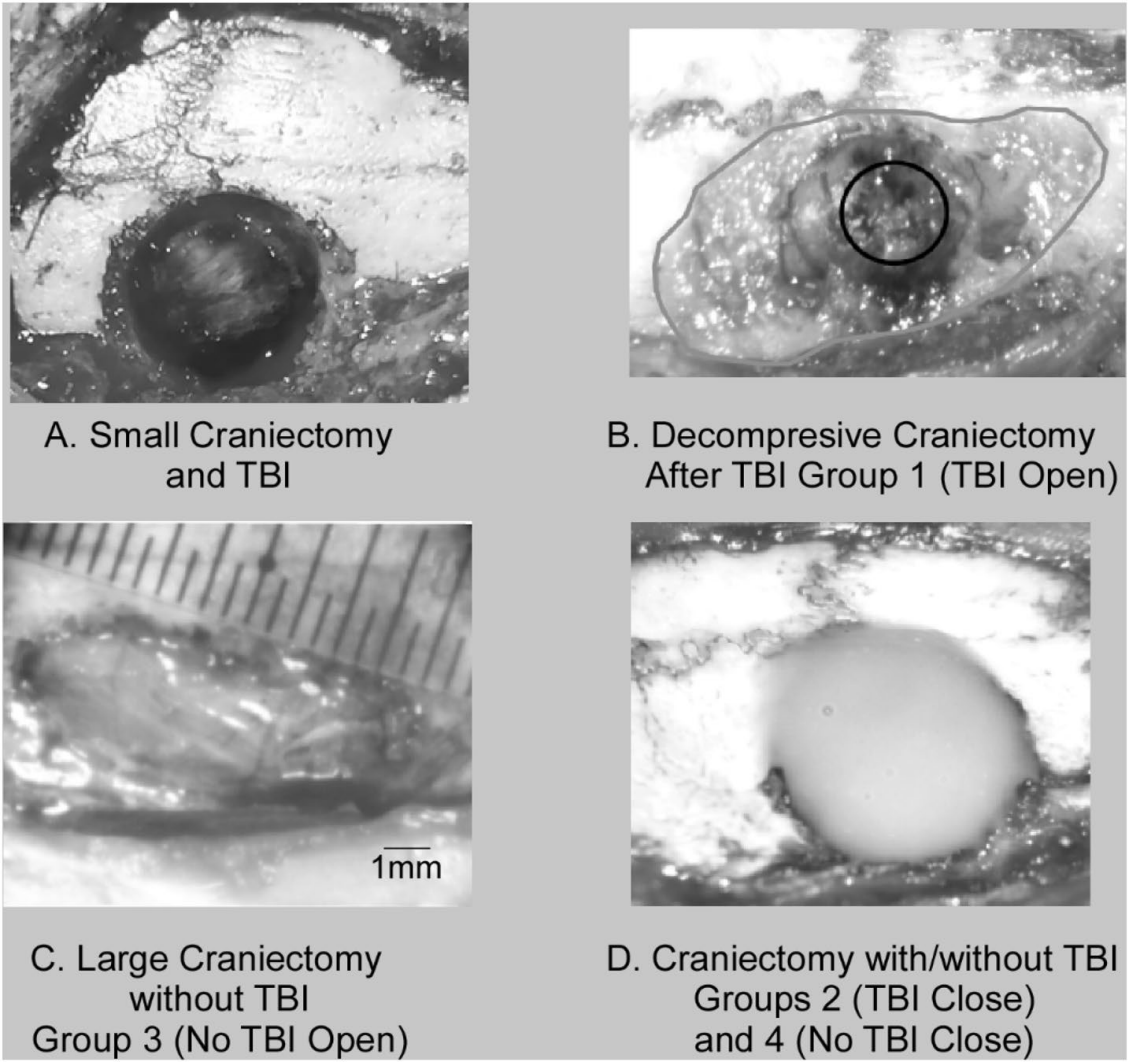
Treatment and Control Groups. Animals were randomly assigned to one of four groups. A small trephination (5 mm diameter) was centered over CFA to access cortex for TBI. **(A)** TBI after small craniectomy. **(B)** TBI Open Skull Group had a decompressive craniectomy following TBI. Black circle indicates impact target over CFA. **(C)** No TBI Open Skull Group, a non-injured control group, had a large craniectomy similar to the TBI Open Skull Group. **(D)** TBI Closed Skull Group had an immediate cranioplasty following 5 mm trephination and TBI. No TBI Closed Skull Group, a non-injured group, had an immediate cranioplasty following the 5 mm trephination over the CFA.

Surgical techniques were similar to those previously described in publications from our laboratory^10–12^. Briefly, rats were anesthetized with an induction dose of 3.0% isoflurane followed by ketamine (80 mg/kg, intraperitoneal) and xylazine (5 mg/kg, intramuscular). Maintenance doses of ketamine (10–15 mg intramuscular) were given as needed. Hair was shaved at the vertex of skull then rats were positioned prone with their head rigidly fixated in a stereotaxic frame. Under aseptic conditions, a midline incision was made over the skull and soft tissue retracted exposing the cranium. Initially, a small cranial defect was performed using a hand-held 5 mm trephination device prior to TBI (Fig. 1a). Special attention was made to leave the dura mater intact. In TBI groups, a commercial controlled cortical impact (CCI) device (Leica Microsystems) was used to perform precise, repeatable brain injury. Readily identifiable stereotaxic coordinates were used to ensure the TBI location was performed at the caudal forelimb area (CFA) of the primary motor cortex in all rats. A 3 mm diameter stainless steel rod with a flat tip, slightly beveled along the perimeter, was used to deliver an impact to exposed cortex with dura intact. The rate of impact was 1.5 m/s delivered at a 2 mm depth below the cortical surface with and impact time of 100 ms. This TBI severity was determined by past publications demonstrating reliable motor deficits with predictable recoverability^10–12^. Open Skull Groups underwent a decompressive hemi-craniectomy procedure (Fig. 1b,c). The boundaries of the hemi-craniectomy were the sagittal (medial), lambdoid (posterior), and coronal (anterior) sutures and the insertion of the temporal muscle laterally. These landmarks allow the creation of a 5 × 10 mm hemi-craniectomy defect. These dimensions are well beyond any published critical rat calvarial defect size thus alleviating the concern for cranial bone regeneration^13,14^. Both Closed Skull Groups underwent immediate 5 mm trephination repair with dental acrylic restoring the normal cranial vault anatomy (Fig. 1d).

### Group assignments

Rats were randomly assigned to one of four groups to assess skilled forelimb performance after craniectomy under four conditions related to TBI and cranial repair. Previous data from a stroke recovery study using similar behavioral assessment found a strong effect size of 2.0; we chose a more conservative effect size of 1.25 with α = 0.05 and power = 0.80 yielding 4 rats per group (G*Power statistical software; Dusseldorf Germany). Two rats were added per group for potential attrition including failure to meet performance inclusion criterion. Two groups of rats received a TBI via a CCI device centered within the CFA of the primary motor cortex. TBI Open Skull Group (n = 5) had a DC immediately following TBI. TBI Closed Skull Group (n = 5) had an immediate cranial repair after 5 mm trephination and TBI. Two additional groups of rats received no TBI. No TBI Open Skull Group (n = 4) had a hemi-craniectomy only. No TBI Closed Skull Group (n = 4) had a cranial repair immediately after 5 mm trephination.

### Rodent behavioral assessment

**Figure 2:**
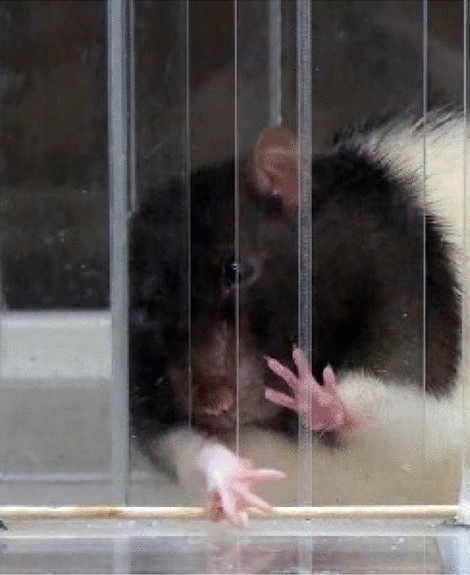
Rats were trained to reach through an opening in a Plexiglas chamber to retrieve small food-pellets located on a shelf placed 3 cm above the floor outside the chamber. A movable wall inside the chamber allowed the rat to use only his preferred forelimb (for TBI groups this was the forelimb contralateral to the TBI). Performance was assessed once per week during two weeks of baseline training, once per week for first 4 weeks after TBI and then once every four weeks up to 16 weeks after TBI.

A standardized pellet-retrieval task was used to assess sensorimotor performance of a skilled reach and grasp task (Fig. 2)^15^. The apparatus was constructed of transparent Plexiglas and measured 19.5 cm long, 8 cm wide, and 20 cm tall. A 1-cm wide vertical reaching slot, extending from the bottom of the chamber to 10 cm in height was centered at the front of the chamber. A 2-cm thick plastic shelf (8.3 cm long and 3.8 cm wide) was mounted 3.0 cm above the floor on the front of the box. Twenty-five 45 mg dustless precision food pellets (Bioserve Inc.; Frenchtown, NJ) were placed in indentations spaced 2 cm away from the window and centered on its edges such that the rat could only reach each indentation with one paw and could not reach the pellets with their tongue. After determining forelimb preference for the task, training was performed prior to surgery by compartmentalizing the chamber with a removable wall to limit reaches to one forelimb only. Each training session consisted of a maximum of 60 trials. Training sessions were conducted 5 days per week until each rat was successful on 70% of the trials for two consecutive weeks. A trial was counted as successful when the rat grasped and transported the pellet to its mouth. Prior to craniectomy and TBI, baseline performance was acquired for two weeks. Post-TBI performance was assessed using video recordings with the experimenter blinded to the group status. Rats were assessed weekly for the first 4 weeks then monthly for a total assessment period of 4 months.

### Ex-vivo magnetic resonance imaging

Four weeks after TBI, two rats (TBI Open Skull Group, n = 1 and TBI Closed Skull Group, n = 1) were sacrificed by cardiac perfusion (4% paraformaldehyde). Heads were immersed in gadolinium prior to MRI scans. MRIs were acquired with a 9.4 T Varian scanner, using a 38 mm quadrature RF surface coil. Images were collected in two separate interleaved spin-echo acquisitions (each with TR/TE = 900/11 ms, Number of averages = 150, matrix size = 384 × 384, field of view = 1.92 × 1.92 cm, slice thickness = 0.25 mm, acquisition time = 14.5 h).

### Statistical analysis

An arcsine transformation was used to normalize the motor performance scores (percentage data) which were analyzed with a repeated measures ANOVA (JMP 11, SAS Inst. Inc.). An index of performance was derived to control for individual differences in baseline motor skills. Successful reach scores were normalized for each rat relative to pre-surgical baseline performance resulting in a ratio: (successful post-surgical retrievals) $\div$ (successful baseline retrievals). An Index of 1 = baseline values (dashed line); an index < 1 = a reduction in successful retrievals relative to baseline; an index > 1 = increased in successful retrievals relative to baseline. An arcsine transformation was used to normalize data for parametric analysis. Specific post-hoc contrasts (Fisher’s protected t) were chosen to minimize the number of comparisons with the intention to interpret whether condition of cranium (open or closed) influenced post-operative performance in injured and non-injured rats.

## Results

### Skilled reaching performance

**Figure 3:**
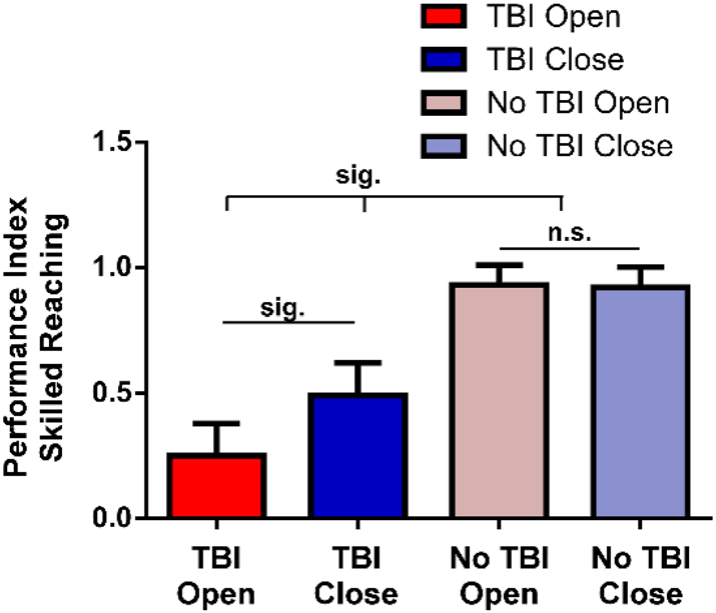
Treatment of cranium following a craniectomy (closed cranial repair vs. open unrepaired skull) was compared between rats having a TBI injury over forelimb motor cortex (TBI Open Skull Group and TBI Closed Skull Group) to rats that had the same craniectomy but no injury (No TBI Open Skull Group and No TBI Closed Skull Group). Rats were assessed over 16 weeks after the craniectomy (once per week for the first month and then once per month for next 3 months). Cranial treatment did not significantly affect performance of the non-injured rats (p = 0.52). Cranial treatment did significantly affect performance of rats with TBI (p = 0.0001). Cranial repair (TBI Closed Skull Group) improved performance compared to rats with non-repaired cranium (TBI Open Skull Group). TBI significantly impaired performance over the 16-week post-operative assessment period compared to the non-injured rats regardless of cranial treatment (p = 0.0001).

**Figure 4:**
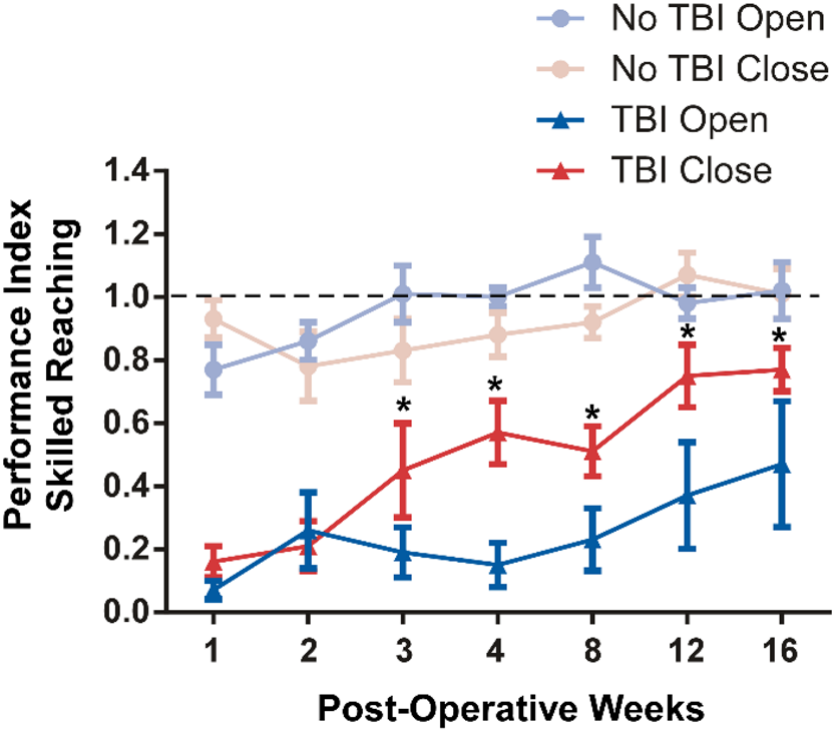
Early cranial repair was beneficial to recovery of motor skills following TBI. Cranial repair improved performance for rats with TBI (TBI Closed Skull Group) beginning 3 weeks after injury. The TBI Closed Group continued to perform better than the TBI rats with open cranium (TBI Open Skull Group) on subsequent assessment days through 16 weeks of recovery. There were no significant differences in performance between the non-injured groups (No TBI Closed Skull Group vs. No TBI Open Skull Group) throughout the 16-week assessment period*.* The horizontal dash line represents a standard index of pre-injury baseline that equals 1 for each animal: pre-surgical success/post-surgical success. Scores under the dashed line indicates motor impairment. The asterisk ‘*’ indicates a group comparison that is statistically significant. P values were derived from post-hoc analysis of the Group by Time effect using Fisher’s protected t-test.

Motor skill performance on the skilled reach and grasp task was analyzed for the four groups and recovery compared with pre-operative assessments. A repeated measures ANOVA revealed significant main effects for Group: F(3,15) = 107.53, p = 0.0001; Time F(6,83) = 7.20, p = 0.0001; and Group X Time Interaction: F(18,83) = 1.92, p = 0.02. Main Group effect contrasts (Fig. 4) show that averaged over time, the TBI Open Skull Group was less successful on the reach and grasp task than the TBI Closed Skull Group (t(1,84) = 8.05, p < 0.0001), the No TBI Open Skull Group (t(1,84) = 16.53, p = 0.0001) and the No TBI Closed Skull Group (t(1,84) = 15.86, p = 0.0001). The TBI Closed Skull Group was less successful than both the No TBI Open Skull Group (t(1,84) = 8.95, p < 0.0001) and the No TBI Closed Skull Group (t(1,84) = 8.27, p < 0.0001). Motor skill performance for the non-CCI groups (No TBI Open Skull Group and No TBI Closed Skull Group), were not significantly different (t(1,84) = 0.65, p = 0.52). (Fig. 3).

Analysis of the interaction effect was conducted for post-CCI weeks 3, 4, 8, 12 and 16 to further assess the effect cranial treatment (open vs. closed) had on performance (Fig. 4). The contrast analyses revealed that cranial repair did not significantly influence performance for the non-CCI groups. There were no significant differences in successful motor skill performance between the No TBI Open Skull Group and No TBI Closed Skull Group at any of the post-CCI assessment days on week-3 (t(1,84) = 0.45, p = 0.66), week-4 (t(1,84) = 0.59, p = 0.55), week-8 (t(1,84) = 0.89, p = 0.380), week-12 (t(1,84) = 0.44, p = 0.66), and week-16 (t(1,84) = 0.02, p = 0.99). Cranial treatment did significantly affect performance for the CCI groups. Cranial repair significantly improved performance for the TBI Closed Skull Group compared to the TBI Open Skull Group on week-3 (t(1,84) = 0.98 , p = 0.0001), week-4 (t(1,84) = 6.67, p = 0.0001), week-8 (t(1,84) = 3.45, p = 0.0001), week-12 (t(1,84) = 5.89, p = 0.0001), and week-16 (t(1,84) = 3.76, p = 0.0003).

### Magnetic resonance imaging

**Figure 5:**
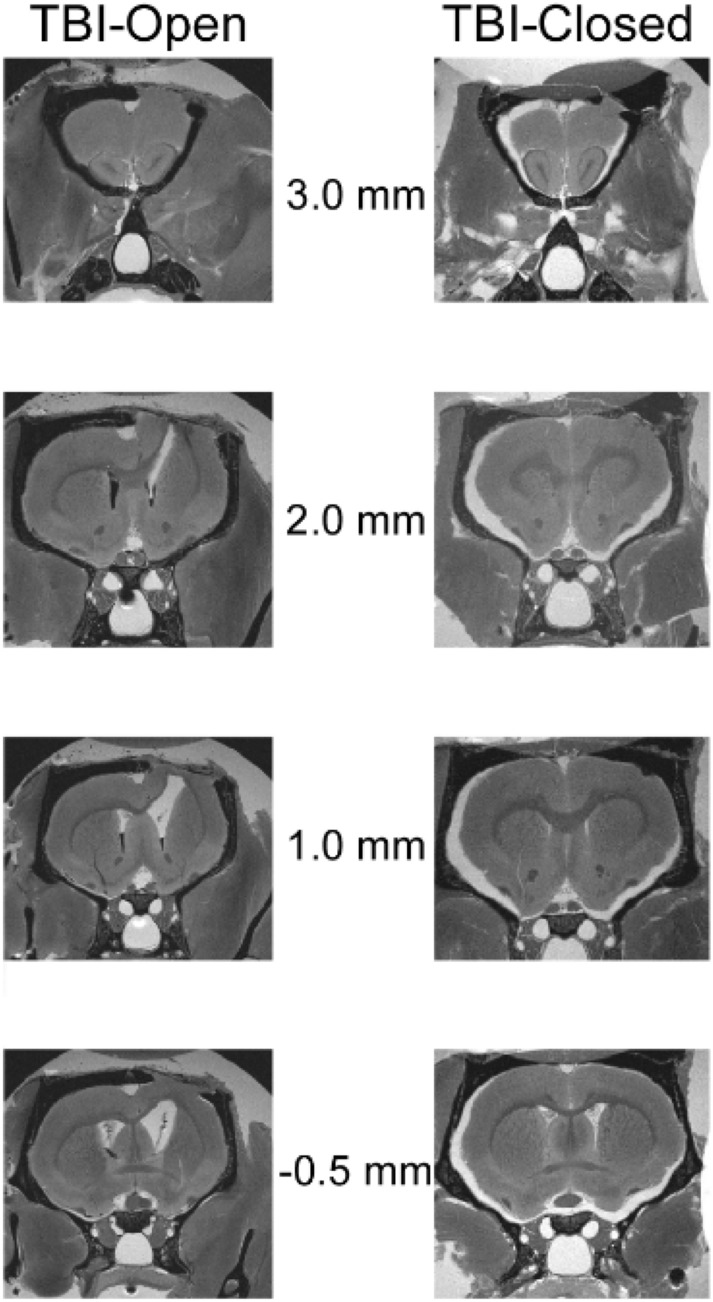
Two rats were scanned post-mortem 4 weeks after a TBI. MRIs were taken with a 9.4 T Varian scanner, using a 38 mm ID quadrature RF coil. To reduce interference between adjacent slices, images were collected in two separate interleaved spin-echo acquisitions (each with TR/TE = 900/11 ms, Number of averages = 150, matrix size = 384 × 384, field of view = 1.92 × 1.92 cm, slice thickness = 0.25 mm, total acquisition time = 14.5 h.). Center column numbers indicate rostro-caudal stereotaxic coordinates relative to the frontal cranial suture line, Bregma. The number between the two columns of MRI figures (3 mm, 2 mm, 1 mm, -0.5 mm) indicate anterior–posterior direction location of the coronal section shown. These are functionally relevant to motor cortex. The rat on the left was given a decompressive craniectomy after TBI (TBI Open Skull Group) while the rat on the right was given a cranioplasty immediately after TBI (TBI Closed Skull Group). In the rat with the decompressive craniectomy (left), cortical tissue herniated out of the cranium whereas cortical anatomy was preserved following open TBI with the cranioplasty procedure (right).

Ex vivo MRI was used to qualitatively assess cortical morphological differences in two rats (one with and one without cranial repair) following TBI. The rat with an unrepaired calvarium (TBI Open Skull Group) demonstrated transcalvarial herniation and gross distortion of the ipsilateral lateral ventricle (Fig. 5). The rat that underwent cranioplasty (TBI Closed Skull Group) had minimal surface herniation and preservation of the underlying neural cortex without substantial cortical deformity.

## Discussion

TBI resulting from open/closed head trauma is a common healthcare problem. Statistics from the US Health Cost & Utilization Project verified that 12,700 decompressive craniectomies were performed in the United States for TBI in 2001^16^. In 2003, direct costs required to treat patients with TBI exceeded $9 billion in the USA alone^17^. Recent global conflicts seen in Iraq and Afghanistan have demonstrated a marked improvement in the management of severe cranial injuries^18^. These neurologic outcome improvements are not the result of improved neurosurgical techniques as decompressive craniectomy techniques remain relatively unchanged for more than 100 years^2^. The predominant factor for the increased survival of severe TBI is the early intervention of decompressive craniectomy at “far forward military medical theaters” within “the golden hour of trauma”^19,20^. These improved outcomes have been the basis for early intervention treatment protocols in civilian TBI management. Data supporting this clinical practice by Friess et al. demonstrated that mice undergoing DC had an 18% reduction in white matter volume compared to 34% to controls (closed skulls)^21^.

Unrepaired decompressive craniectomy defects have been well described to cause neurologic symptoms of SoT that are reversible with cranioplasty^22^. SoT remains a poorly understood neurologic condition for several reasons. First, limited animal models exist that mimic the human condition completely. Second, the symptoms overlap with other conditions encountered in TBI subjects such as post-concussion syndrome (PCS) and post-traumatic stress disorder (PTSD). What makes SoT a unique neurologic condition is that the neurologic deficits are completely reversible immediately following a cranioplasty procedure to restore the native cranial vault anatomy. A systematic review by Ashayeri et al. demonstrated that SoT occurs in 60% of neurotrauma patients studied^8^. Furthermore, this study concluded that SoT lacks exact characterization and deserves further investigation and is supported by a similar meta-analysis by Annan et al. who found similar findings^7^.

To date, DC remains the neurosurgical procedure of choice to treat severe TBI despite its known limitations. As such, a better understanding of the risks and benefits of this commonly performed procedure is necessary. Although SoT is reversible with a cranioplasty procedure, patients would be better served if SoT was prevented. This becomes more important when we consider the variability of time between DC and cranioplasty that TBI patients experience^23,24^. Factors that impact this discrepancy include surgeon and operating room availability, patient co-morbidities and availability secondary to ongoing neurorehabilitation, and third party payment provider requirements.

Animal TBI models investigating DC are limited; however, previous rodent studies support an association between an open or unrepaired cranial vault and adverse outcomes. Szczygielski et al. showed that DC increases brain lesion volume and exacerbates functional impairment in mice. Similar to our study, DC was performed in the same surgical procedure (one hour later); however, they utilized a closed TBI in contrast to our open injury^25^. Zweckberger et al. have also investigated the impact of DC on brain swelling and secondary brain damage 24 h after TBI^26^. Using mice, a “large” open CCI (DC sized cranial defect) was utilized to create an initial TBI and an immediate cranioplasty was performed using “conventional tissue glue” (Histoacryl, B. Braun, Bethlehem, PA). A secondary DC was performed 1, 3 and 8 h following CCI by removing the tissue glue (no further bone removal). They demonstrated less brain edema 24 h post TBI as a result of secondary DC at the 1- and 3-h time points. Their differing outcomes in comparison to both Szczygielski and our study are likely impacted by their method of large open TBI, non-rigid cranioplasty material (tissue glue), and early brain edema analysis (24 h post-TBI). MRI images from our current study (72 h post-TBI) demonstrate that the injured brain beneath the unrepaired skull expands and herniates with the loss of the physical cranial barrier. In addition, the lateral ventricle was distorted as a result of unrestricted brain herniation resulting from the hemi-craniectomy. In comparison, MRIs of rats with a TBI and an immediate cranioplasty demonstrated less significant cortical injury and better neural preservation at the TBI site. These findings support the idea that the cranial vault not only serves as a protective barrier in the setting of TBI, it is also necessary to maintains cortical anatomy which may be beneficial in neural recovery.

The aim of this study was to demonstrate motor deficits in rats undergoing DC independent of TBI. Rats that had a TBI followed by immediate DC (a common TBI treatment) performed worse than those that had an immediate cranial repair. In addition, as hypothesized, rats who had no TBI but did undergo DC performed also demonstrated worse motor outcomes in comparison to those who had a TBI and immediate cranial repair. These persistent deficits in the unrepaired rat calvarium (independent of TBI) mirror those seen in humans with SoT. In a recent publication by our group, 143 human subjects who underwent DC demonstrated a 28% incidence of SoT^9^. Of these 40 subjects with SoT, 70% demonstrated complete functional return on average 4.3 days following cranioplasty. The rapid and complete reversal of symptoms does not fit the current understanding of either PCS or PTSD making SoT a unique neurologic condition. Thus, SoT is often overlooked in the treatment of TBI patients.

Previously our group has demonstrated that motor deficits in a mouse animal model are reversible with a cranioplasty procedure^10^. A beam walk test was used to observe “footfaults” on both the ipsilateral and contralateral hindlimbs. A CCI delivered at the CFA immediately adjacent to hindlimb area caused no ipsilateral hindlimb deficits; however, footfaults were statistically more likely in the contralateral hindlimb in mice that did not undergo cranioplasty as expected. Our current study provides similar results by demonstrating persistent forelimb motor deficits using a rat model. Rats left with an unrepaired cranial defect had worse motor performance recovery compared with those that underwent immediate cranioplasty following TBI. Both our current study and our human data demonstrate an association with unrepaired cranial defects and decreased motor performance^9^. *Ex-vivo* MRI demonstrated preserved cortical anatomy in the rats that underwent cranial repair following TBI which likely accounts for the improved motor performance when compared to TBI rats that underwent hemi-craniectomy.

There are several limitations of this current study. First, study group sizes were limited and utilized male rats only, negating the known gender differences in TBI recovery and neuroplasticity^27,28^. In addition, our study utilized an open TBI procedure using a CCI device. Rodent TBI literature is plentiful using a plethora of TBI mechanisms including: CCI, fluid percussion, and weight drop/impact (I/A) acceleration^29^. Our study utilized CCI to ensure that the TBI was standardized to same CFA location using the same width, velocity, and depth thus controlling for injury amongst animals in the study. In humans, TBI is not controlled and is often closed resulting from blunt trauma, thus injury patterns vary greatly. Future studies should compare DC and cranioplasty in other injury models and animal species. Another limitation of the current study is that the cranioplasty was performed immediately following TBI as supported by our past rodent TBI publications^10,11^. This was done to avoid a second surgical procedure and possibly further trauma or confounding factors. As such, we chose a CCI to the CFA whose severity is known to cause reversible focal motor deficits. Although possible, more severe TBIs would likely result in non-reversible deficits or death. Finally, dental acrylic was used as our cranioplasty material secondary to its accessibility, ease to work with, and fast onset of solid, non-flexible consolidation. The acrylic was placed over a small 5 mm trephination defect and was unlikely to impact the underlying brain parenchyma. MRI imaging demonstrated no adverse effects of the use of dental acrylic as demonstrated by the two animal subjects with available imaging.

## Conclusion

Large unrepaired defects from treatment of TBI in humans are well documented to be associated with reversible neurologic deficits termed syndrome of the trephined. In the current study, rats who underwent decompressive craniectomy following TBI demonstrated prolonged, statistically significant motor deficits when compared with rats who underwent immediate cranial reconstruction.
